# Impact failure in two silicates revealed by ultrafast, *in situ*, synchrotron X-ray microscopy

**DOI:** 10.1038/s41598-020-67086-3

**Published:** 2020-06-25

**Authors:** N. K. Bourne, W. U. Mirihanage, M. P. Olbinado, A. Rack, C. Rau

**Affiliations:** 1 Department of Mechanical, Aerospace and Civil Engineering, The University of Manchester M13 9PL, Manchester, UK; 2 Centre for Matter under Extreme Conditions, University of Manchester at Harwell, Diamond Light Source, Harwell Campus, Didcot, OX11 0DE UK; 3 Department of Materials, The University of Manchester, Manchester, M13 9PL UK; 4 ESRF-The European Synchrotron, Grenoble, France; 5 Diamond Light Source, Didcot, OX11 0DE UK

**Keywords:** Mechanical engineering, Glasses, Applied physics, Characterization and analytical techniques

## Abstract

To travel safely behind screens that can protect us from stones and hail, we must understand the response of glass to impact. However, without a means to observe the mechanisms that fail different silicate architectures, engineering has relied on external sensors, post-impact examination and best-guess to glaze our vehicles. We have used single and multi-bunch, X-ray imaging to differentiate distinct phases of failure in two silicates. We identified distinct micromechanisms, operating in tandem and leading to failure in borosilicate glass and Z-cut quartz. A surface zone in the amorphous glass densifies before bulk fracture occurs and then fails the block, whilst in quartz, fast cracks, driven down cleavage planes, fails the bulk. Varying the rate at which ejecta escapes by using different indenter tip geometries controls the failed target’s bulk strength. This opens the way to more physically based constitutive descriptions for the glasses allowing design of safer, composite panels by controlling the impulses felt by protective screens.

## Introduction

Glass has been used in windows since Roman times. However, the propensity of silicates to fracture has encouraged an understanding of elastic stress fields developed by Hertz and his successors^1^. Griffith advanced understanding of failure for perfectly brittle solids, but even so predicting failure of this class of materials still represents a challenge since their microstructures are both filled with flaws and are not fully dense so that they can compress under load^2^. It is only recently that we have been able to understand the micromechanics by which this amorphous substance physically responds to impulsive loads^3,4^. Understanding these mechanisms allows the design of a structural, transparent often laminated window that can protect from fragments, hail or stones impacting cars, aircraft or panes in buildings^5^. When forces acting on an amorphous material exceed its strength, fracture generally initiates at a flaw to define the end of elastic behaviour^6^. Inelastic processes start when failure is initiated on the loaded surface of the material where residual stress concentrations are typically concentrated^7^. At this time, cracks propagate at a speed determined by the stress level, and these may accelerate to a limit which peaks at the Rayleigh wave speed in the material^8–10^. Of course, such loading imposes high stresses in the impact zone, and the response of glasses to high pressures has been studied in some depth, building on the work of Bridgman^11^. The most demanding loading is provided by the shock impulse that applies the extreme stresses at the highest rates^12–15^. In a particular regime of stresses, fracture is delayed in glasses behind the propagating front^16–18^. An interesting result is that the failure initiation is further retarded in borosilicate glass in these experiments^19^. Upon impact, a structure must absorb the strain applied until failure processes remove resistance or the impulse applied by the impactor is absorbed^15,19^. For a propelled stone or hail particle, this loading time is typically only a few microseconds, but in the case of amorphous materials like glass at these low stress amplitudes, structural failure occurs on a timescale an order of magnitude slower^20,21^. Yet when impact speeds are accelerated, failure processes occur much faster and the impactor may penetrate before its impulse is consumed^22,23^. Indentation offers a well-documented technique that can be used to cross the loading rates to connect static analyses to the dynamic regime^20,24–27^. Typically, experimental campaigns measure loads to failure and image fracture morphology after unloading (*ex situ)* to deduce micromechanisms during loading. While white light photography during *in situ* measurements yields information on the evolution of zone boundaries, the opacity of fractured glass removes information from within failing zones themselves. However, using monochromatic X rays allows the density of structure within failing zones to be resolved. These processes are typically simulated with empirical descriptions within finite element codes that degrade the macroscopic strength in their constitutive models for brittle solids^28,29^. This has met with limited success, since the fracture (localisation) occurs on a length scale commensurate with that of mesh elements over which properties are assumed to be homogeneous. To advance these short comings, we show here key experiments that identify distinct mechanisms operating within the failure process using continuous and single-bunch imaging, at the European Synchrotron Radiation Facility (ESRF)^30–32^.

All solids, including amorphous materials, show the evolution of unsteady stress states through localisation, failure and compaction as a material accommodates strain and transits to steady state deformation. Delayed failure is an important attribute of brittle material response, and this phenomenon plays a critical role in the protection of key components in systems and vital structures^33^. In previous work it has been possible to measure stress and wave speeds for these evolving, failed states using optical imaging^34,35^. To observe the morphology of fracture in glasses and ceramics, one typically needs *ex-situ* X-ray tomography^36–39^ and optical and electron microscopy^31^ if representative volumes of the sample material remain intact. Before the advent of single bunch radiography, it was difficult at best to track and identify the evolution of the fracture field in real time for impact driven cracks using X rays. Further, using optical imaging it was not possible to observe failure occurring within the bulk, since observations were typically made through opaque, failed *surfaces* in the experiments conducted^26,35,40^. Thus we have little information about density changes in the processes observed unless the target recovers without failure and can be examined *ex situ*^41^.

It is well known that fracture kinetics in glasses is strain rate sensitive and that reducing temperature ensures that materials showing any degree of inelasticity behave in a more brittle manner^21,25^. In parallel work to this, crack propagation at intermediate strain rates has been observed using radiography at the APS synchrotron^37^. Here Kolsky bar loading, and an adapted three-point bend test, were used to drive cracks into silicates and boron carbide. These results relate single fractures to bulk stress intensity factors, illustrating the limitations of applying a macroscopic engineering parameter to local failures. In the shock regime, and for failure within distinct regions of influence, the connection is more readily made between failed and as-received strength. Thus, in this work we describe synchrotron-based radiography to quantitatively define the evolution of these impact states whilst observing bulk fracture occurring in the glasses. We have previously investigated features of these phenomena using white-light photography^33,42^, and we now extend these data using synchrotron-based radiography to couple understanding of materials’ physics with characterisation of the geometry and kinetics of failure across regimes.

Previous work has published high-speed optical imaging of impact-induced failure in glasses^35,40,42^. This has spanned planar impact experiments to image failure waves, that travel behind propagating shock fronts, in parallel with fast sensors to define the state. Other works have identified failure from different projectile geometries including spheres^25,26,35^. In all cases, there is a danger that reflected pulses from free surfaces either end the failure initiation process or fail the glass at the target surface. Both these effects obscure effects in the bulk when interrogated with white light since it will not transmit when surfaces open by more than half a wavelength of illuminating wave. Further, insufficient confinement will result in a partial failure that required suitable confinement in this work to prevent release in the glass before processes in the impact zone had completed. This work investigates features of the dynamic failure process, impacting a full density and an open structure silicate. X rays allow observation of operating mechanisms in the critical first moments of compression before penetration begins.

The failure of the silicates was observed in real time using X-ray imaging at the ESRF beamline ID19^43^. During these experiments, the ESRF storage ring was operated in a dedicated four-bunch operation mode. Here, four distinctive electron bunches were circulated in the storage-ring with a current equivalent to 10 mA per bunch. The bunch full-width-half-maximum length was 140 ps and this made a realistic exposure time lower than 200 ps. The revolution frequency of a single bunch is determined by the storage-ring circumference and is calculated to be *ca*. 2.8 µs. Thus, with 4 bunches, it is possible to acquire images every *ca*. 700 ns. This machine-operation mode was instrumental in fixing the frequency of the *in situ* imaging experiments, matched to the operating failure mechanisms in these glasses. In most experiments phase contrast imaging was used in order to better resolve propagating waves. In experiments where we aimed to investigate density variation, a monochromator allowed us to directly image density variation. More details are presented in the methods section.

**Figure 1:**
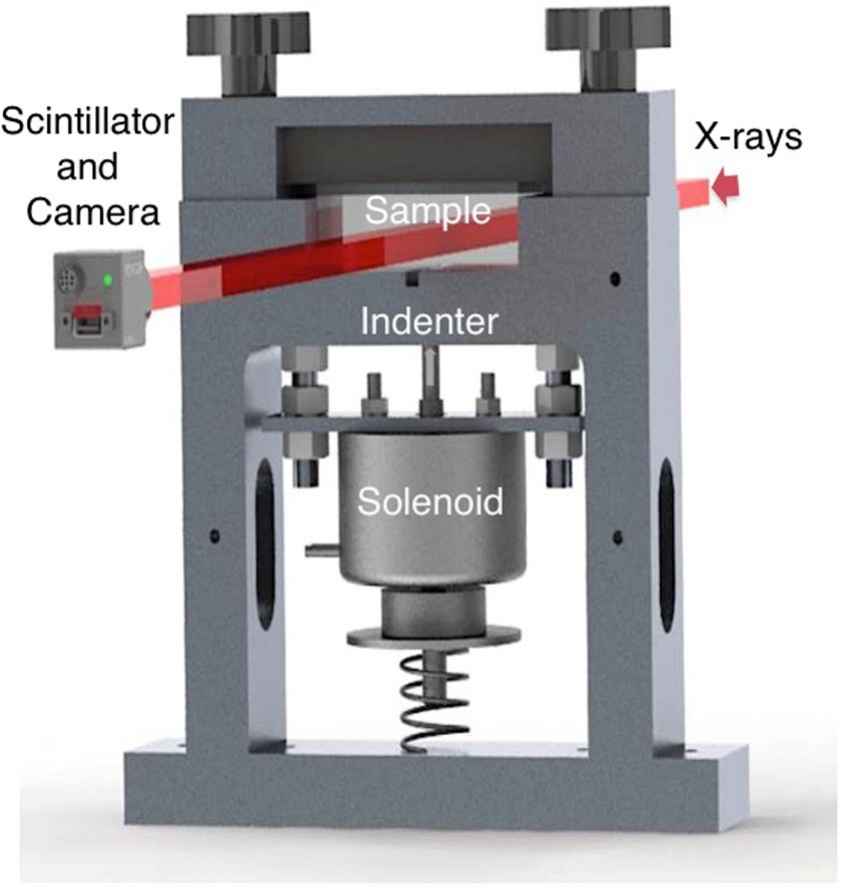
Schematic of the portable indentation device showing the imaging configuration adopted for these tests.

For observation of *in situ* failure, samples were indented using different tip morphologies and fracture propagation radiographed to build up an overview of the failure processes using a specially designed fast indentation device (Fig. 1 and see methods). Silicate plates were impacted onto a ground, flat face to drive a divergent failure front into the target. Impact experiments were conducted on borosilicate glass and crystalline, Z cut quartz samples and X-ray image sequences with an inter-frame time of 1.404 μs, we recorded for each trial.

**Figure 2:**
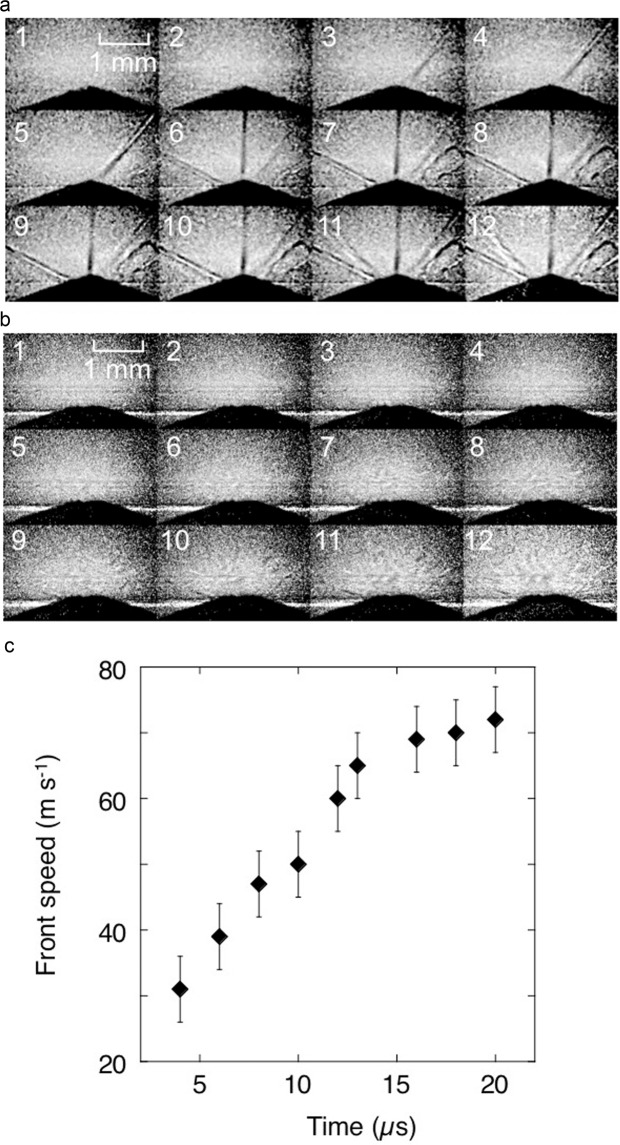
Impact of a triangular prismatic indenter onto (**a**) quartz (**b**) borosilicate targets. (**c**) Evolution of the damage region front velocity in borosilicate glass. X-ray image sequences have an inter-frame time of 1.404 μs.

Figure 2 shows image sequences for both borosilicate and Z-cut quartz targets, each failing after impact from a prismatic-nosed indenter. These sequences interrogated the initiation phase of bulk failure in the material with frames acquired every 1.4 µs by the camera. This prismatic nose geometry loaded the material down a single impact axis and launched a cylindrical shock front into the target, observed end-on in these sequences.

The development of the impact zone in Z-cut quartz (Fig. 2a) shows fast, localized failure initiating a limited number of isolated cracks (at *ca*. 30 and 60° to the impact axis), which are activated along principal shear planes discussed in the previous section. Since there is no densification of material at the impact point, the material fails an order of magnitude faster than its borosilicate counterpart and energy is not dissipated in compression or multiple failure processes that diffuse the applied impulse. It will be seen that it takes *ca*. 20 µs for the failure front tip to propagate within the field of view in borosilicate, while it has developed in less than 2 µs, for a leading crack propagating within quartz.

**Figure 3:**
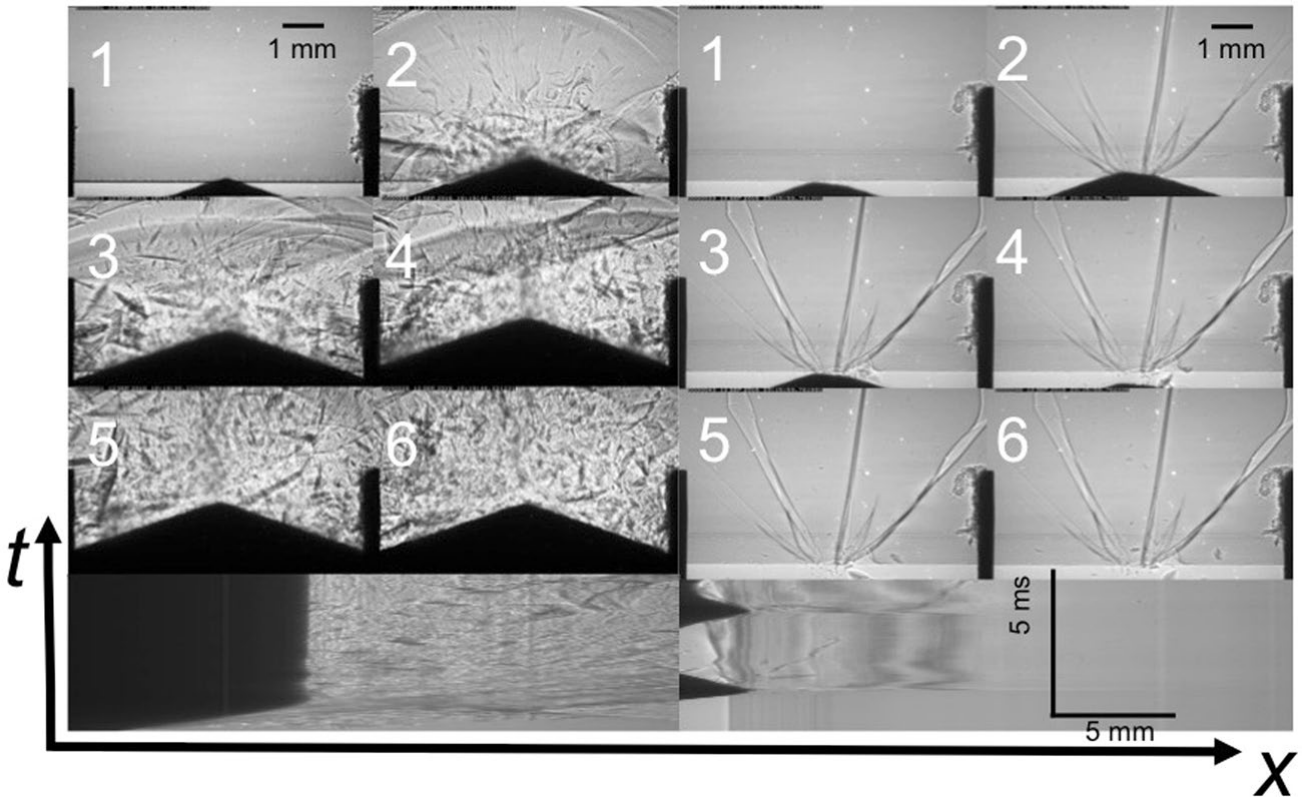
Impact of a triangular prismatic indenter. BS sequence to right and quartz to left. Conditions as for those of Fig. 2. The upper part of the figure shows frames from radiographic sequences whilst below each is a streak image for each impact with dark regions showing the indenter and grey-scale reflecting the density of damaged regions down the central axis of the target. Interframe time *ca*. 68 µs.

A radiographic sequence showing the failure of borosilicate glass by the indenter is shown in Fig. 2b) at the same rate as above. Here there is a significant time for which the indenter rests at the impact face without significant fracture being apparent. Since we have monochromatic illumination, this lower density, failed region is a zone of compression that develops as indentation occurs. The density change evident is small since there are multiple fractures present and each is only separated by tens of nm in each case^44^. Nevertheless, the failed zone propagates, and eventually allows the indenter to penetrate, the failed region driving fracture. At much later times fractures propagate into a large volume to accommodate the strain imposed and, following displacement of failed material, the target bulk strength is reduced allowing the indenter to penetrate and pass through (Fig. 3). The front locus is plotted in Fig. 2c). and achieves a speed of up to 80 m s^−1^, around a quarter of the Rayleigh wavespeed for this material. The error bars reflect repeated measurements across several repeat sequences and uncertainties in the position of the densified zone. In the later stage, the whole block fails through multiple cracks, initiating and then propagating outwards from the indenter position. It is known that borosilicate glass, failing under a macroscopic, one-dimensional loading, has a strength of *ca*. 2 GPa^33^. In uniaxial strain experiments the glass loses half of its strength in *ca*. 1 µs^18,19^. Here, such loading will act upon the initiation region that occupies a few mm from the impact face. The stress at the indenter tip is an order of magnitude less here than is required to fail the glass in plate impact experiments (in which the target is inertially confined), but in the latter, failed material cannot flow outwards and its bulk strength is thus maintained for a longer time. Here, the geometry of the tip lets failed material expand and allowing the indenter to enter. In this case, significant tensile stresses develop once the waves from first contact travel far enough to access free surface beyond the contact zone and these fail the material here and begin to degrade the failed strength of the material accessed by the tip.

As shown in Fig. 2c), the damage zone in the borosilicate glass has a front that accelerates to *ca*. 70 m s^−1^ from an initial velocity in the indentation zone of around 30 m s^−1^. This reflects the release of material from the first densification processes observed in the initial contact stage. However, in quartz the initial crack velocity down a line of principal shear in the crystal exceeded 500 m s^−1^ while the components of the failed zone do not release during the time for which the impulse is applied. This is an indication of the pseudo-instantaneous initiation of failure and the strong effect of localization in the activated slip planes in the crystal and, as expected, the Y plane is initiated first since it has lower fracture toughness than the X and Z planes^38^.

The progression of damage, with the same geometry indenter, imaged at slower framing rate, is presented in Fig. 3 for both the borosilicate and the quartz. Both experiments are typical of the set of repeat shots conducted for each impact with the triangular, prismatic indenter. The upper part of the figure shows frames from radiographic sequences taken on each material, recorded with phase contrast filtering to highlight failure features. Each is numbered left to right; top to bottom with an interframe time of *ca*. 68 µs. A borosilicate sequence is shown to the left, while an equivalent impact onto quartz is shown to the right-hand side. Below each sequence is a streak image for each impact (a continuous plot of the impact state evolving along the impact axis as a function of time), with dark regions showing the indenter and the grey scale reflecting the density of damaged regions down the central axis of the target. The triangular indenter used in this test fails the borosilicate tile and penetrates the fragmented material as discussed above. In the case of the quartz, there are localised cracks initiated from a principal flaw in the impact zone, which travel down the surface down the principal shear directions^37^. The magnetic field driving the indenter can be controlled to allow further impacts to occur, and in the case of the quartz target, the indenter tip returned after the first impact and loaded the failed region a second time (see streak image bottom right). This second loading did not isolate further damage zones. Rather it extended fractures already in place although the indenter still rebounded from the target.

In the case of the indentation into BS glass (top left), first impact can be seen in Frame 1, where penetration has already begun to induce a region of densification. The process does not initiate propagating fractures down trajectories of principal shear in this case. Local failures can be seen at separations of order hundred micron within the impact zone discussed above. By the second frame, the compressed zone has driven a series of local failures into the tile and the indenter has penetrated several millimetres into the target. A surface wave front, driven from the impact zone and decelerating as it disperses in its travel, can been seen at the top of the frame which has travelled *ca*. 10 mm by this stage of the impact. This densification front further reflects from the steel surround that confines the glass plate, but there is, nevertheless, no damage evident behind these wave fronts. By frame 3, around 150 µs after impact, damage propagates into the tile, and fragmentation is evident in a region that flows forward at tens of m s^−1^. This stops after frame 5 when the indenter has lost all of its momentum. The streak image, taken down the impact axis through the indenter tip and beneath the framing sequence, shows in the first moments the slowing and arrest of the indenter and the drive of the fracture front ahead of it. The arrest of the penetrator indicates the end of the failure region with only of isolated fragments visible crossing the axis thereafter.

In the first impact onto quartz, the indenter propagates four symmetrical cracks along different principal shear planes with 30° between each of the planes. The rebound and second impact induces limited isolated cracks, while the second impact opens these further, isolating fragments and dissipating impulse from the impact. The streak image for the quartz (bottom right) clearly shows the double impact of the rebounding indenter onto the target surface. There is a perturbed region, localized to the first 10 mm of the target, that corresponds to the extent of the steel mounting blocks and within this, local effects from interactions from reflected waves occur.

During the study, experiments were repeated with the same materials and indenter types, whilst other parameters were kept constant to show reproducible failure modes within the targets. Comparison of those image sequences shows clear, repeatable behaviours found over single and multiple impacts, of which those shown here are representative examples.

**Figure 4:**
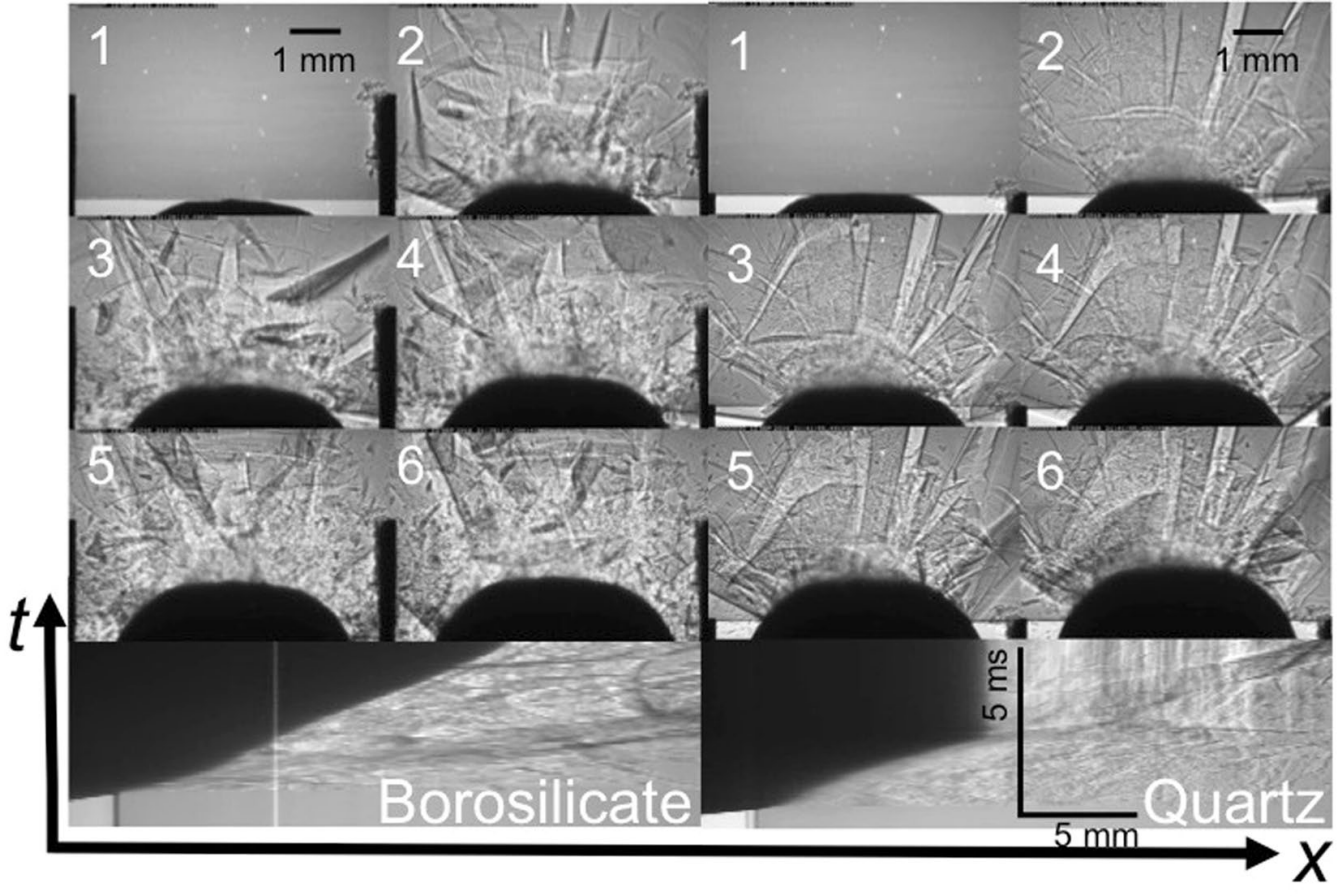
Impact of a spherical indenter. BS radiographic sequence to right and that for quartz to left. The upper part of the figure shows frames from radiographic sequences whilst below each sequence is a streak image for each impact with dark regions showing the indenter and grey-scale reflecting the density of damaged regions down the central axis of the target. Interframe time of *ca*. 68 µs.

To further illustrate the effect of the secondary unloading on the failed strength, experiments using spherical-nosed indenters were recorded, and a typical pair of sequences are shown in Fig. 4. Such *in situ* impact experiments have been conducted previously and interrogated using high-speed optical photography by Chaudhri^26,35^. In this case, the borosilicate fails in clearly visible expanding fronts that slow the indenter, but do not stop it. This geometry allows further penetration to occur as material is more efficiently expelled from the impact zone after failure, which lowers the strength of the fractured target. In later stages, the failure behaviour is qualitatively similar to that seen for the prismatic indenter (Fig. 3) with fragmentation occurring in a similar manner. However the failure processes take less work to complete and thus the impulse is sufficient to drive the indenter through the fragmented zone. There is qualitative similarity with previous observations of glass failure after impact from a spherical projectile^45–47^.

As previously, there is less damage observed in the quartz target than in the borosilicate one, although whilst it began to penetrate, the indenter was stopped within the target. Again failure down planes of principal shear is observed immediately impact occurs, but in this case the nose further intrudes into the target activating more defects on the impact face. This causes a family of cracks to propagate that locally fail material in the impact zone. Further, since the failed material can more easily escape from the impact face, the indenter can penetrate at least some distance into the target. In all cases, failure development is ended when the impulse from the indenter is spent as seen in the borosilicate case above.

In all of these experiments, it is clear that failure occurs in discrete stages and at different rates. These failure modes operate on different timescales which require access to ultrafast, single-bunch X ray imaging to differentiate between the local compression and fracture, and the result bulk strength degradation phases that determine late time penetration and ejecta. The indenter supplies an impulse that has done work on the microstructure, leading to different failure modes in the two silicates. In the initial contact, cracks are driven down the planes of lowest fracture toughness in the crystalline quartz, thus failure spreads into the bulk quickly through fracture down planes of principal shear within the crystal structure. However, fracture is delayed in borosilicate in the first moments as a densification zone develops for some time before a homogeneous fracture field is driven out into the bulk. The first stages in both cases are driven by mechanisms operating in the local state within the impact zone itself, whilst later penetration occurs orders of magnitude slower at the macroscopic scale of the structure insulted. In particular, we observe that the high theoretical strength of quartz can be overcome at modest levels, and in certain indenter nose geometries, because of the ability to trigger failure at defects in the flawed surface zone in targets subject a divergent flow field.

This work has used two modes of fast, synchrotron imaging to observe a suite of processes that contribute to the macroscopic failure of silicates. These new, ultrafast imaging techniques have allowed observation of the evolution of failure across multiple length and time frames and have shown a particular feature of dynamic failure not observed using optical photography. Two different microstructures have been shown to exhibit different initial failure modes operating to fail brittle solids. This is reflected in the varied damage modes observed, leading to a range of failed strengths, in glasses impacted in engineering applications^47^. Our loading device applies selected impulses in a controlled impact geometry to test the response and interfaces directly with fast, *in situ* X-ray radiographic, diffraction and spectroscopic experiments. The ability to resolve crack tip movement and the propagation of defect fronts in these chemically similar, yet mechanically distinct brittle materials, has identified micromechanisms operating in the first stages of impact and shown that they reduce the bulk properties of the glass to determine the engineering response to penetration at the macroscale. This shows the reasons for delayed inititation of fracture seen previously in borosilicate glass and points the way to identify controlling mechanisms that drive failure in brittle structures under impact. Further, these observations inform debate on the interpretation of bulk strength under various loading regimes with application to studies concerning formation of planetary bodies through impact and accretion^48^. Finally, improved constitutive models will allow the design of layered, composite panels to respond to impulses experienced by ultrafast projectiles at a range of scales.

## Methods

For this work the ID19 beamline at ESRF was operated with two U32 undulators in series (nominal minimum gaps of 11.1 mm and 11.5 mm). This configuration delivered a mean energy of approximately 30 keV with a usable polychromatic X-ray energy spectrum largely confined to an energy range between 20 keV and 50 keV. Image acquisition was conducting using propagation-based phase contrast and the imaging detector was therefore placed at *ca*. 10 m distance. This consisted of a 250 µm thick, LuAG scintillator coupled to either HPV-X2 camera with 400 ×250 pixels per frame (Shimadzu Corporation, Japan) or a pco.dimax camera (PCO AG, Germany) with 624 ×16 pixels per image. The Shimadzu camera was used only for single bunch imaging with an 8 µm effective pixel size. The HPV-X2 camera was used to capture 128 images with 10 bit greyscale and was used to obtain fast image acquisition without data transfer problems. The pco.dimax was employed to produce a single frame, integrating light from several X-ray flashes, and delivering image sequences with relatively longer time spans, at *ca*. 14.5 kHz frame rate with an effective pixel size of 11 µm^49^.

For single bunch imaging, capturing the indentation and crack propagation within the camera’s recording window was a critical consideration. This required the camera aperture to be temporally synchronized with the X-ray flashes from each electron bunch. Thus, the initial triggering signal was passed to the indenter, and a proximity sensor detected the position of the shaft (±20 µm accuracy) sending a 5 V DC pulse to a digital delay generator to trigger image capture in synchronization with the X-ray flashes. To get the optimized synchronization, images were acquired every 1.404 µs and the camera aperture was controlled to have a single frame for each X-ray bunch^31^.

### Fast indentation device

A specially designed, portable indentation device, capable of initiating failure across a range of material classes, was designed such that it might trigger from signals generated by fiducials from the synchrotron (Fig. 1). The drive for the device was applied using a commercially available electrical solenoid (Ledex Low Profile 6SFM). An approximate force of 900 N was delivered by the solenoid to a series of indenter geometries that reached velocities exceeding 1 m s^−1^ at impact. The solenoid shaft was mounted in line with the freely moving indenter tip so that different, hardened-steel, tip geometries could be inserted for each experimental sequence. The sample stage allowed targets of rectangular cross-section, and a range of thickness varying from 0.5 mm – 15 mm, to be placed in the device. The target blocks in this case were samples of dimension 75 mm square impacted onto a ground surface in their centre. The thickness of the targets had a effective lateral dimension such that surface regions failed several ms before damage was released at the free surfaces. Two open sides allowed the X-ray beam to pass uninterrupted through the sample holder to collect transmission image across an adequate field of view. Two of the indenter geometries used in the experiments can be seen in Figs. 2–4. These were in each case a prismatic indenter designed to load down the beam axis to allow fast framing down the beam axis and a hemisphere of radius 5 mm. The prismatic form was 10 mm at its base and included an angle of 142° to mimic in 2D the Berkovich geometry^50^. The indenters for all the experiments were made from hardened, martensitic steel (440 A). Little blunting of the tip was observed between shots but indenter tips were replaced between experiments in this work.

### Materials

Borosilicate glass (BS) and Z-cut quartz were used as representative, silicate sample materials. Material properties for the two are shown in Table 1. The defect population controls the dynamic properties of a material with their size and separation fixing the strength and the kinetics of localisation and failure under load. All experiments described here were conducted at ambient temperatures and there were no effects observed due to irradiation of the glass targets compared with experiments done using high-speed visible light photography prior to the experiment. There are two classes of defect present within the BS glass - pores and microcracks within the bulk. The pore fraction results from a population of air bubbles entrained within the melt sheet that were of order tens of microns in dimension but separated from one another by distances of millimetres. Microcracks had a similar volume density and separation. Material properties for the BS are presented in Table 1. α-Quartz is a brittle, hard, dielectric that is a dominant mineral in geophysics and used in a series of industrial applications^51^. It has trigonal structure and is loaded in this work along the crystal *c* axis. Various workers have determined values for the fracture toughness down different crystallographic directions using static indentation techniques^52,53^. Typical values are presented in Table 1. The fracture toughness is greatest down the loading direction and takes lower values in the other principal directions. In all cases the value is, of course, greater than the value for the borosilicate glass.

**Table 1:** Material properties for the two silicates tested. Data taken from references^3,26–28^.

	Borosilicate (BS)	Z cut Quartz
*ρ* (±0.05 g cm^−3^)	2.23	2.65
*E* (GPa)	73.1	X (112-0) 78.3
		Y (101-0) 78.3
		Z (0001) 104.2
*µ* (GPa)	30.4	31.1
Poisson’s ratio *ν*	0.20	0.40
*c*_L_ (±0.01 mm µs^−1^)	5.56	6.37
*c*_S_ (±0.01 mm µs^−1^)	3.45	4.71
*K*_1C_ (MPa m^1/2^)	0.80	1.15 (Z)
		0.97 (Y)
		0.85 (X)
HEL (±0.5 GPa)	4.0	12.0

Both these materials have been observed to exhibit greater compressive strengths in dynamic rather than static loading. In the extreme case, with the fastest rising impulses, shock loading of the two materials has shown a range of values in their elastic limits (in a macroscopic uniaxial strain state) for the materials under load^19^. Here the target is assumed to fail over a front where strain rate is maintained at values of 10^6^ s^−1^ and higher. The Hugoniot elastic limit in such experiments is *ca*. 12 GPa, which initiates failure in compression within the shock front^54^. Here the strain imposed is somewhat different, with a tensile component in the propagating wave as the impact front travels outward from the indenter tip. Nevertheless, the state in the first moments will not reach such stresses and any failure thus will be dominated by local fracture around defects on the impact face. It is these that these new techniques will be uniquely able to capture using the fast imaging fielded in this work at ID19.
